# ‘Pscore’ - A Novel Percentile-Based Metric to Accurately Assess Individual Deviations in Non-Gaussian Distributions of Quantitative MRI Metrics

**DOI:** 10.1101/2023.12.10.571016

**Published:** 2023-12-10

**Authors:** Rakibul Hafiz, M. Okan Irfanoglu, Amritha Nayak, Carlo Pierpaoli

**Affiliations:** 1. Laboratory on Quantitative Medical Imaging, National Institute of Biomedical Imaging and Bioengineering, Bethesda, MD; 2. Military Traumatic Brain Injury Initiative (MTBI2 – formerly known as the Center for Neuroscience and Regenerative Medicine [CNRM]) Bethesda, MD; 3. The Henry Jackson Foundation for the Advancement of Military Medicine, Bethesda, MD

**Keywords:** Normative Distribution, Individual Deviations, Skewness, Diffusion MRI, Zscores, Extreme Value Bias

## Abstract

**BACKGROUND:**

Quantitative MRI metrics could be used in personalized medicine to assess individuals against normative distributions. However, conventional Zscore analysis is inadequate for measurements with non-Gaussian distributions.

**PURPOSE or HYPOTHESIS:**

Demonstrate systematic skewness in diffusion MRI (dMRI) metrics. Propose a novel percentile-based method, ‘Pscore’ to address this and document its performance on a publicly available dataset.

**STUDY TYPE:**

Cohort

**POPULATION:**

961 healthy young adults, the Human Connectome Project (HCP)

**FIELDSTRENGTH/SEQUENCE:**

Siemens 3T ‘Connectome Skyra’ scanner, spin-echo diffusion echo planar imaging (EPI)

**ASSESSMENT:**

The dMRI data was preprocessed using the TORTOISE pipeline. Average values within 48 regions of interest (ROIs) were computed from various diffusion tensor (DT) and mean apparent propagator (MAP) metrics. For each ROI, percentile ranks across participants were first computed to generate ‘Pscores’– which normalize the difference between the median and a participant’s value with the corresponding difference between the median and the 5^th^/95^th^ percentile values.

**STATISTICAL TESTS:**

ROIwise distributions were assessed using ‘Log’-transformation, ‘Zscore’ and the ‘Pscore’ methods. The distributions and percentages of extreme values (>95^th^ and <5^th^ percentile boundaries) were also compared across all ROIs comprising the overall white matter. Bootstrapping was performed to test the reliability of Pscores in small samples (N=100) using 100 iterations.

**RESULTS:**

The dMRI metrics demonstrated systematic skewness, leading to skewed ‘Log’-transform and ‘Zscore’ distributions. Zscores showed extreme value biases, which were strongest for the Propagator Anisotropy. ‘Pscore’ distributions were symmetric and robustly maintained 5% extreme values in both tails, even for 100 iterations in small, bootstrapped samples.

**DATA CONCLUSION:**

The inherent skewness observed for dMRI metrics preclude the use of conventional Zscore analysis. The proposed ‘Pscore’ method accurately estimates individual deviations in skewed normative data. Although the HCP dMRI data was showcased, Pscores offer a general solution, even for smaller databases with non-Gaussian distributed values of neuroimaging and clinical measurements.

## INTRODUCTION

Neuroimaging studies in clinical research typically rely on group level analyses delineating summary outcomes that differentiate a patient cohort from a group of healthy controls. However, in clinical practice, there is a need to assess individual patients. This is typically done by building a model to evaluate the individual subject against a normative sample.(1–4) Quantitative MRI metrics can provide normative data against which individual deviations can be assessed. Therefore, once the accuracy and reliability are verified, quantitative MRI metrics could become very relevant for clinical assessment of individual patients.

Individual assessment against a normative distribution is often performed using Zscores in clinical(5) and neuroimaging(2,3) paradigms. However, a thorough assessment on the distribution that generates the normative dataset itself, is rarely performed. An example is the Gaussianity and homogeneity of variance, a fundamental assumption for parametric models. Deviation from these assumptions result in biased statistical inferences, for example, in neuropsychological test scores.(6) Quantile regressions(7) have been adopted to mitigate this by generating normative distributions at specific percentiles. It offers a solution based on distributions at selective percentile/quantile ranks and avoids the use of conventional ‘mean’ regression model which would be biased for asymmetric distributions.

The problem is pervasive even for quantitative MRI metrics. In a pilot study (N = 48), we have recently shown prominent deviation from normality and heavy-tailed distributions of several diffusion tensor (DT)(8,9) and mean apparent propagator (MAP)(10) metrics.(11) For example, the Mean Diffusivity (MD) had a positively skewed distribution, while Fractional Anisotropy (FA) and Propagator Anisotropy (PA) showed a negatively skewed distribution. In our small sample, these non-Gaussian features were inherently related to the diffusion characteristics of water in the brain, and not originating from heterogeneity in the underlying population as it has been previously reported(1,4) in large scale public datasets. For example, for the UK-Biobank data,(12) Fraza et al fit a warped gaussian model to compensate for the skewness and kurtosis in the normative data before using Zscores to assess heterogeneous individuals.(1) Another approach popularly used to address skewness is log transformation, however, it is known to worsen the issue of skewness(13) and lead to inaccurate and biased inferences.(14) Our initial assessment showed that even when comparing individuals within the normative sample, Zscores show a negative bias in extreme values for negatively skewed distributions and, on the other hand, a positive bias in extreme values for positively skewed distributions.(11) Therefore, comparing patients against such biased distributions using Zscores will wrongly place some of them in the extremities of the distribution and introduce false positives.

In this study, we first test if the systematic skewness previously observed in our pilot study using dMRI metrics can be reproduced in a larger sample from a homogeneous group of healthy young subjects with a narrow age range. Secondly, we propose a percentile-based novel quantity, called ‘Pscore’, to accurately estimate an individual’s deviation from the central tendencies of a normative distribution, and compare its accuracy against two popular normalization methods. The ‘Pscore’ method was also tested on the data from our pilot study and proved quite robust even for a small sample of 48 controls. Here, we fully test and validate this method systematically, using dMRI data from the Human Connectome Project (HCP).(15)

## MATERIALS AND METHODS

### Participants

We used the HCP S1200 series, which has at least 1113 participants with 3T MR imaging data available from a pool of 1200 young adults (age range: 22–35 years). The data includes structural MRI, functional (fMRI) and diffusion MRI (dMRI) data along with behavioral and genetic testing. For this study, we included only dMRI data from 971 individuals used in one of our previous works.(16) Ten of these participants were ‘36+’ years of age, and they were excluded to keep the sample more homogeneous within the 22–35 years range. Thus, the effective sample included neuroimaging data from 961 participants (53% females). All participants were scanned on the same equipment, using the same protocol and we encourage the reader to read the HCP reference manual(17) for more details.

### Diffusion MRI Protocol

The Connectome 3T Skyra scanner was used to acquire diffusion MRI images across 6 runs in a full session. Each run was approximately 9 minutes and 50 seconds long, with three different gradient tables and each table was acquired with two phase encoding directions – right-to-left and left-to-right. Each gradient table consisted of six b = 0 s/mm^2^ acquisitions and approximately 90 diffusion weighting directions. The diffusion weighting was done across three b-shells – 1000, 2000 and 3000 s/mm^2^, each with equal number of acquisitions per run. The imaging parameters include – spin-echo EPI sequence with TR = 5520ms, TE = 89.5ms, flip angle = 78°, refocusing flip angle = 160°, Field of View (FOV) = 210×180, matrix = 168×144, slice thickness = 1.25 mm, 111 slices acquired at 1.25 mm isotropic resolution, a multiband factor of 3 and echo spacing = 0.78ms.

### Preprocessing

We used the TORTOISEV3 pipeline,(18) to process the dMRI data, because Irfanoglu et al., had shown significant improvement in the dMRI metrics using this pipeline compared to the released version of the HCP dataset.(16) Following, we briefly describe the different stages of pre-processing – *(a) Denoising* was performed using a model-free noise mapping technique proposed by Veraart et al.,(19) with a kernel radius of 3. *(b) Gibbs-ringing correction* was performed using the subvoxel-shift method,(20) which showed improvements even without introducing additional imperfections. For the *(c) Inter Volume Motion and Eddy Current Correction –* we first applied a MAP-based model, as it is independent of shelled-data and accurately extrapolates the unseen q-vector signals. All diffusion weighted images (DWIs) were aligned to the ideal b = 0 s/mm^2^ image. As a final step, step *(c)* was run once more, except, in this instance, the synthesized and the slice-transformed real images were used. These steps were iterated over until convergence was reached. *(d) Susceptibility-induced distortions* – Acquiring data at high resolution (like the HCP) causes severe EPI distortions. We applied the DRBUDDI approach,(21) which is a blip-up and blip-down distortion correction technique with excellent performance.(22) Besides using the b = 0 s/mm^2^ image, DRBUDDI also incorporates diffusion tensors and relies on an undistorted T2W structural image. However, the T2W image from the HCP data was not compatible with DRBUDDI, therefore, we used a machine learning-based technique – SynB0-DisCo,(23) to generate a structural image that fit well into the DRBUDDI paradigm. *(e) Gradient nonlinearity correction* – The HCP data comes with “gradwarped” DWIs and gradient-deviation tensor images. Effects of inter-volume motion are not considered when a single gradient-deviation tensor is used for all DWIs. Therefore, we also computed the voxelwise B matrices, to take these effects(24) into consideration. *(f) Signal drift correction* – Owing to the long scan time, signal drift was observed in the HCP data, therefore, they were corrected using a method proposed in.(25) *(g) Normalization and template generation* – The processed data were generated at both the HCP isotropic resolution of 1.25 mm and at the 1 mm resolution of the processed T1W image. A diffusion tensor-based registration was applied, and an atlas was generated(26,27) using the 1 mm resolution data and the DWIs were warped on to the template space using non-linear transformation.

### Diffusion MRI Metrics

We generated voxelwise maps for four DT metrics – fractional anisotropy (FA)(28), mean diffusivity (MD), axial diffusivity (AD) and radial diffusivity (RD). We also generated voxelwise maps for five MAP metrics – propagator anisotropy (PA), return to axis probability (RTAP), return to origin probability (RTOP), return to plane probability (RTPP) and non-Gaussianity (NG).(10) Each of these metrics carry useful quantitative information about the various diffusion behavior of water in the brain.

### Quality Control Assessment

Quality Control assessments are vital for neuroimaging applications, especially those that involve complex interpolations and multiple preprocessing steps. We took several steps in assessing each dMRI metric map for each subject. All dMRI maps registered to the study template were first visually inspected. The maps from 961 subjects were checked for misregistration and abnormal warping using an in-house custom-built script that generates contour lines of all maps over corresponding subject’s T2W image in the template space. Subjects that failed the quality control assessment were checked again and appropriate steps were taken to correct the registration issues. The datasets that could not be salvaged despite this extensive correction pipeline, were removed from further analysis leaving an effective sample of 960 and 912 participants for the DT and MAP metrics, respectively.

### Regions of Interest (ROIs)

To reduce the number of tests and focus on specific WM regions, we used a set of regions of interest (ROIs) for the current study. The ROIs were inspired by the John Hopkins University (JHU) WM ROIs,(29) however, they were manually redrawn(30) on an average DT brain template built from the HCP dataset(26,31) to avoid issues with left/right structural asymmetry that have been reported for the original JHU ROIs.(32) Moreover, the JHU ROIs were defined in a scalar map and uses scalar-based registration, which tend to have misregistrations.(26) We used a tensor-based registration, which provides better alignment. The JHU ROIs are quite large and therefore, we drew the ROIs to ensure they are ‘within’ the tracts/structures to reduce partial volume effects. ROI labels were created and the average DT and MAP metrics were computed for each subject across all ROIs using ITK-SNAP.(30) Figure A shows the ROIs in a detailed montage across all three orthogonal planes for better visualization and assessment. Please refer to Table S1 in the supplement for more details on these ROIs.

### The Pscore Method

The first step in computing Pscores is generating percentile ranks for each participant. They were computed within each ROI using the following formula –

[2] $$p_{ij}=\frac{n_{NS\leq x_{ij}}}{n_{NS}}\times 100;\;i=1,2,3\ldots N,\;j=1,\;2,\;3,\ldots M$$

where, $x_{ij}$ represents the average diffusion MRI metric value of the $i^{th}$ individual for the $j^{th}$ ROI, and i representing 1…N(960) participants, and j representing 1…M(48) ROIs, respectively. $n_{NS\leq x_{ij}}$ represents the number of participants within the normative sample having a value $\leq x_{ij}$. The denominator $n_{NS}$ represents the total number of participants in the normative sample.

After the percentile computation, each participant’s position in the ROIwise distribution is considered to assess which side of the tail they are represented in. This is identified by the difference between the participant’s metric value and the median of the distribution. This difference is then normalized with either the difference between the median and the 5^th^ or the 95^th^ percentile edge value, depending on the participant’s position. The following equations depict how a Pscore is computed on either side of the median:

[3] $$P_{5_{ij}}=(-)1.645\;\frac{d_{x_{ij}}}{d_{5}};\left\{\begin{array}{l} d_{x_{ij}}=x_{ij}-m_{j}|x_{ij}<m_{j}\\ d_{5}=m_{j}-x_{j_{5}}|x_{ij}<m_{j} \end{array}\right.$$

[4] $$P_{95_{ij}}=(+)1.645\;\frac{d_{x_{ij}}}{d_{95}};\left\{\begin{array}{l} d_{x_{ij}}=x_{ij}-m_{j}|x_{ij}>m_{j}\\ d_{95}=x_{j_{95}}-m_{j}|x_{ij}>m_{j} \end{array}\right.$$

where, $d_{x_{ij}}$ is the difference between $x_{ij}$ and the median of the distribution, $m_{j}$ for the $j^{th}$ ROI. If the metric value of the $i^{th}$ participant, $x_{ij}<m_{j}$, then $d_{x_{ij}}<0$, which means the participant is located on the left-hand tail between $m_{j}$ and the 5^th^ percentile edge value, $x_{j_{5}}$ of the distribution. The corresponding denominator $d_{5}$ is computed as the difference between $m_{j}$ and $x_{j_{5}}$. On the other hand, $d_{x_{ij}}>0$, when $x_{ij}>m_{j}$, indicating the participant’s position on the right-hand tail between $m_{j}$ and the 95^th^ percentile edge value, $x_{j_{95}}$ and the denominator $d_{95}$ is computed as the difference between $x_{j_{95}}$ and $m_{j}$. Both $P_{5_{ij}}$ and $P_{95_{ij}}$ takes on the polarity of $d_{x_{ij}}$, generating the negative and positive scores, respectively. The ratios of these differences are then scaled by |1.645| representing the Zscore value corresponding to the 5^th^ and 95^th^ percentiles of a normal distribution. This brings the Pscores to the scale of Zscores and makes them comparable.

### Analysis

We analyzed the data ROIwise using three normalization techniques – ‘log transformation’, ‘standardized Zscore’ and the ‘Pscore’ method proposed in this study. Histogram distributions of the raw data were used as a reference to compare the distributions from these three methods. For each histogram plot, the mean and median lines were added to assess deviations from the mode and misalignment of these moments. A normal density curve was fit on top of the histograms to assess which normalization method was closer to a Gaussian distribution. This leads to four figures per ROI for each dMRI metric, generating 1728 figures in all (4 figures × 48 ROIs × 9 dMRI metrics). It is difficult and beyond the scope of this study to show all these figures.

Therefore, to summarize and simplify, we took the Zscore and Pscore values from the entire sample across all ROIs and decomposed them into two single column vectors. For example, for a DT metric such as FA, each ROI had Zscores from 960 healthy individuals. This would create a vector of 46080 Zscores (960 Zscores × 48 ROIs) and similarly, for Pscores. Please note, for the MAP metrics, since data from 912 individuals survived the quality control step, the vector had 43776 Zscores (912 Zscores x 48 ROIs). These vectors were then used to generate two distribution plots – one for Zscores and the other for Pscores. The process was repeated for each DT and MAP metric. Please also note that the ‘log transformation’ method was not included in this step, as we already mentioned the issues with this approach, and it performs very poorly at the ROI level (see Figure B and Figures SA, SB). Additionally, log-transformed values are intrinsically incompatible with Zscores and Pscores. Since the Pscores are in the same scale as Zscores, they can be compared and assessed together. This was to showcase which of these two normalization techniques showed a statistical imbalance in the data spread across the entire WM and over the entire sample.

More importantly, for each metric, this step also helped quantify the extreme value bias present in the tails of these distributions. A normal Z-distribution would have 5% of extreme values above the 95^th^ percentile (Z = 1.645) and below the 5^th^ percentile (Z = −1.645). We quantified and tabulated the number and percentage of these extreme values for Zscores and Pscores for all dMRI metrics. In the presence of an extreme value bias, the balance of 5% will be altered in the tail ends of these distributions. This helped showcase which of these normalization methods maintained a systematic bias at the tail ends that may lead to inaccurate assessment of individuals whose values are in the tails of the distribution.

To test how Pscores perform on smaller samples, we performed bootstrapping on the pool of HCP participants (N = 960). We ran 100 iterations, each time, randomly selecting 100 participants and repeating the Zscore vs. Pscore comparison at the overall-WM level discussed above. We also performed 20 iterations, independently on the dMRI metric that showed the strongest skew and extreme value bias across all WM ROIs. This was done for brevity and showcasing the reliability of the Pscore approach on the metric least expected to conform to Gaussianity.

To highlight the level of extreme value bias in Zscores compared to Pscores, we generated heatmaps, similar to the ones shown in our pilot study.(11) It is an intuitive way to visualize the level and direction of the bias at the individual level. The heatmaps were generated across all ROIs for the entire population, as well as for a single iteration of the random sampling consisting of 100 participants to reduce the complexity of showcasing 92160 observations (46080 Zscores + 46080 Pscores). Since the pattern is consistent even in small samples, it can be shown with much better clarity for 100 participants with 9600 observations (4800 Zscores + 4800 Pscores). It also helped showcase the advantage of using Pscores in small sample neuroimaging data.

## RESULTS

### ROIwise Comparison of Distributions Across Different Methods

Figure B shows some examples of distributions from the normalization techniques tested, within the Body of the Corpus Callosum (BCC). Please note, distributions from only four dMRI metrics are shown for one ROI, however, assessment was made across each ROI per dMRI metric (not shown here to avoid cluttering). The ‘raw’ (light gray) distributions demonstrate the presence of skew in the dMRI metrics which cause the mean, mode and median to misalign. FA and PA show a negative skew, while MD and RTPP show a positive skew. The mean (dashed green) appears separated from the median (dashed red) and mode (tallest bar). Comparing the histograms with a fitted normal density curve (dashed purple) also illustrates the deviation from a Gaussian distribution. These patterns are also clearly visible for the ‘log’ transformed (dark gray) and ‘Z-score’ (light red) distributions at varying levels. Such skews introduce extreme value bias in the tails (see Assessing Extreme Value Bias later). On the contrary, for all Pscore distributions, the mean, mode and median appear nicely aligned and they tend to fit closely to the normal density curve. Therefore, compared to the other normalization methods tested, and particularly, the standardized Zscore approach, Pscores provide a more symmetric and unbiased distribution per ROI. Please see the supplement for more examples from other dMRI metrics across other ROIs (Figures SA, SB).

### Zscore vs. Pscore Distributions Across All ROIs

Figure C shows the Zscore and Pscore distributions for each dMRI metric, generated from the data across all WM ROIs. In general, Zscores show an overall imbalance around the 0-line for all metrics (less prominent for AD). The percentage (%) of values above/below the 0-line are provided on either side of each distribution plot. An imbalance is indicated by a ‘> 50%’ and ‘< 50%’ value on either side of the 0-line. The Zscore distribution of FA (top left, light red), for instance, shows an overall imbalance with 51% of the total observations above 0 and 49% below it. This means there are at least 460 (1% of 46080) more positive Zscores than negative Zscores in the distribution. The mean (green vertical dashed line) and the median (red vertical dashed line) also appear not aligned. This pattern is observed to be progressively worsening for NG (54% positive) (bottom right, light red) and PA (59% positive) (middle, second row, light red), respectively. The rest of the DT and MAP metrics showed the opposite trend for Zscores, with ‘> 50%’ of total observations below 0. For example, both MD and RTAP had 51% of all the observations as negative values. Therefore, MD had at least 460 and RTAP had at least 437 (1% of 43776) more negative Zscores than positive Zscores in their respective distributions. It was worse for RD, RTOP and RTPP, because each of their distributions had 52% negative values, indicating twice as much negative values than MD and RTAP. All Pscore distributions (light blue), on the other hand, maintained a balanced distribution (50%) of positive and negative values around the 0-line for every single dMRI metric. The mean and median lines align, and the histograms attain a closer fit to the normal density curve (solid blue) compared to the Zscores (solid red).

### Assessing Extreme Value Bias

Table1 quantifies the bias present in the tail ends of the Zscore distributions and the correct balance of extreme values from the Pscore distributions. For each dMRI metric, it shows the number of extreme values above the 95^th^ percentile (P_>95_(%), Z > 1.645) and below the 5^th^ percentile (P_<5_(%), Z < −1.645) for both these normalization methods. Except for AD, where the imbalance is negligible but still < 5%, Zscores from every other dMRI metric shows a bias in extreme values in the left or right tails. For example, FA, NG and PA had progressively larger imbalance and negative skew (see Figure C), and as a result, a subsequent increase in the imbalance of the percentage of extreme values. Compared to ‘P_>95_(%)’, the ‘P_<5_(%)’ values in these three metrics were higher by approximately 1.2 (4.9/4.1), 1.9 (5.4/2.8) and 5.8 (5.8/1) times, respectively. On the other hand, MD, RD, RTAP, RTOP and RTAP showed an imbalance and positive skew for Zscores (see Figure C), leading to a positive bias. All ‘P_>95_(%)’ values for these metrics were > 5%, while all ‘P_<5_(%)’ values were < 5%. Contrarily, for the same dMRI metrics, the ‘P_<5_(%)’ and ‘P_>95_(%)’ values for Pscores maintain a balanced 5% extreme values in both tails.

### Bootstrapping the HCP Sample

Figure D shows a heatmap of PA generated from one iteration of a random sampling of 100 HCP participants. The heatmap intuitively demonstrates any systematic bias of extreme values in individuals (columns) across multiple ROIs. It also highlights any bias present in ROIs (rows) across the normative sample. The heatmap of Zscores (top) shows a large negative bias in extreme values with much less positive extreme values, whereas the Pscore heatmap (bottom) shows a proper balance of positive and negative extreme values, as expected of a normative sample. For simplicity, we showcase a heatmap comprising only 100 participants.

Table2 shows similar quantities as Table1, except for PA, with 20 iterations of random sampling 100 HCP participants. The rationale to highlight PA was because it showed the highest systematic skews and extreme value bias among all dMRI metrics (see Figure C and Table1). For all 20 iterations, Zscores of PA show large negative biases (all ‘P_<5_(%)’ > 5% and all ‘P_>95_(%)’ < 2%) with on average > 4.3 (6.1/1.4) times more negative extreme values than positives. Contrarily, Pscores robustly maintain 5% extreme values in both tails for all 20 iterations. Please refer to Table S2 in the supplement for a summary of the bootstrapping assessment of all dMRI metrics across 100 iterations.

## DISCUSSION

Using the large-scale, high-resolution HCP dataset, we showed a systematic skewness in DT and MAP metric distributions derived from dMRI. We also showed that these skews lead to extreme value biases when building a normative dataset using Zscores. We proposed a novel percentile-based metric – ‘Pscore,’ which is immune from this bias and attains a closer fit to a Gaussian distribution both at the ROI level and the overall WM. We further documented the robustness of this method for smaller samples (N = 100) using bootstrapping. We replicated our previous findings from a study conducted on 48 controls(11) and systematically validated this method in creating an unbiased normative database using the HCP.

Our assessment on the metric distributions showed that diffusivity (e.g., MD, RD) tends to be positively skewed whereas anisotropy (e.g., FA, PA) tends to be negatively skewed. It is difficult to unequivocally identify a primary source for these findings, however, given the narrow age range of the examined population, it is unlikely to be due to aging related effects that have been invoked for large-scale public datasets(1,4,33). A possible explanation for these skewed distributions could be related to the presence of a small percentage of fast diffusing water molecules with isotropic diffusion behavior that have been reported in healthy brain parenchyma.(34) This fast diffusing water compartment has the same diffusion signature as CSF partial volume contamination. CSF contamination within an ROI, will lead to higher mean diffusivity (positive skew) because the diffusivity of CSF is at least four times higher than that of the brain parenchyma.(35) On the other hand, the anisotropy of CSF is virtually 0, which would cause the anisotropy of the WM tracts to be lower (negative skew). These considerations suggest that skewed values may be inherently driven by underlying biological characteristics of the brain and not be necessarily related to heterogeneity in the demographics. One of our rationales for choosing the HCP young-adult sample was its well-balanced homogeneity in demographics (e.g., age and sex).

Our findings may be of particular interest to investigators assessing individual deviations against a skewed normative database. Normative models are typically built from very large samples and often incorporate Gaussian process models.(1,4) This can be counter intuitive as scaling becomes a big challenge(4) with Gaussian processes, as sample sizes get much bigger. Some methods have been proposed based on machine learning to address this issue, but with some caveats of pre-tuning and modeling complexity.(36–38) Indeed, one can simply assess a quantity, e.g., an individual’s height by comparing against the percentiles generated from a sufficiently large population. However, aside from other confounds, neuroimaging studies are often limited by the sample size (median N = 23, according to(39)) and typically, consist of < 100 participants. In a healthy cohort of 48 controls, we have previously shown that normative data generated from dMRI metrics deviate from normality and suffer the consequences of biased inferences when Zscores are used.(11) The study also underscored a key advantage of the ‘Pscore’ approach – its ability to reliably estimate individual deviations in small samples.(11) Therefore, Pscores offer a practical solution to studies with more realistic sample goals (e.g., N = 50 – 150). It can help investigators to accurately assess individuals by building site specific normative databases from neuroimaging data that do not conform to Gaussianity.

Deviation from Gaussianity leads to biases in the peripheral centiles of a normative distribution and inaccurate inferences.(1) Zscores are commonly used for such inferences, and we have established the biases that manifest if the issue of Gaussianity is not addressed. There are approaches that have been adopted to overcome non-Gaussian traits due to sample heterogeneity, implementing various modes of Bayesian linear regressions.(1,40) The ‘Pscore’ approach addresses the issue of Gaussianity at the level of the data distribution itself. By leveraging the median as reference and using percentile ranks, it avoids the bias from the asymmetry in the distribution. Therefore, it can be an important addition to the various arsenal of contemporary normalization techniques. Pscores provide a simple platform to normalize raw data and scale them to standardized Zscores derived from a normal distribution. This is significant, because conforming to normally distributed Zscores facilitates statistical relevance to inferences made on individuals. It allows a meaningful interpretation of an individual’s position, especially when considering the extreme ends of a distribution.

When an individual, such as a patient, is expected to be at the extremities of a normative distribution, it can be considered as a rare event, as most individuals are not expected to be positioned there. Extreme value theorem(41) and its subtypes(42) can prove useful in such cases by fitting asymmetric curves on extreme value distributions derived from such rare events. This has been applied in neuroimaging applications to assess individuals from highly heterogeneous clinical cohorts using normative modeling.(1,4) The process involves generating normative probability maps (NPMs)(43) and computing a Zscore per brain region/voxel that normalizes the difference between the true normative and predicted individual response with their corresponding variances. These Zscores are then usually used to run univariate statistical tests with multiple comparison adjustments. Our expectation is that Pscores can prove very useful in these applications and provide more accurate estimations for extreme events compared to Zscores.

## LIMITATIONS

The Pscore computation relies on percentile ranks of every participant in the normative sample. Therefore, the sample size may be a limiting factor on the confidence and precision of the percentile edges. For example, in our pilot analysis of 48 controls,(11) the smallest resolution of each percentile was 2.083 or ~ 2% (100/48). Since the 5^th^ and 95^th^ percentile edges were used, there was some statistical uncertainty to the exact values representing these percentiles. However, the Pscores still maintained a balanced number of extreme values in the two tails. This issue obviously recedes as sample sizes get bigger, as we see in the current context (N = 961), where the percentile resolutions were much finer (~ 0.1%) and allowed very precise edge measurements. Furthermore, the limitation can be overcome even with smaller samples if precise and even percentile edge measurements can be performed. Take the bootstrapping step for instance, the percentile resolutions were exactly even at 1% (100/100) and Pscores robustly maintained 5% extreme values over 100 iterations for all dMRI metrics (see Table S2). Another limitation for Pscores is the selection of the percentile edges. We used the 5^th^ and 95^th^ percentile edges because they are statistically relevant and allow at least 10% of extreme values in the whole distribution. Choice of a more extreme percentile edge may affect the normalization accuracy. An appropriate alternative could be to look at the area under the curve, instead of point values at percentile edges. That way the computation will be done on a continuous scale, and it also preserves the feature of Pscores to address the issue at the level of the distribution, without making any prior assumption of Gaussianity. This is part of our future project using various samples of multimodal neuroimaging data.

## CONCLUSIONS

In conclusion, although the ‘Pscore’ approach was tested on dMRI metrics, the method itself is not data selective. The ‘Pscore’ method instead looks at the data by virtue of its distribution. Therefore, any study analyzing skewed data with the goal to perform normative predictions on individuals, can incorporate this method. This can include data from clinical, research and neuroimaging paradigms. Particularly for neuroimaging, other modalities, such as structural volumetric and functional MRI can utilize the ‘Pscore’ approach to assess individuals because metrics from these paradigms also suffer from small sample limitations and non-Gaussian distributions.

## Supplementary Material

Supplement 1

## Figures and Tables

**Figure A.**
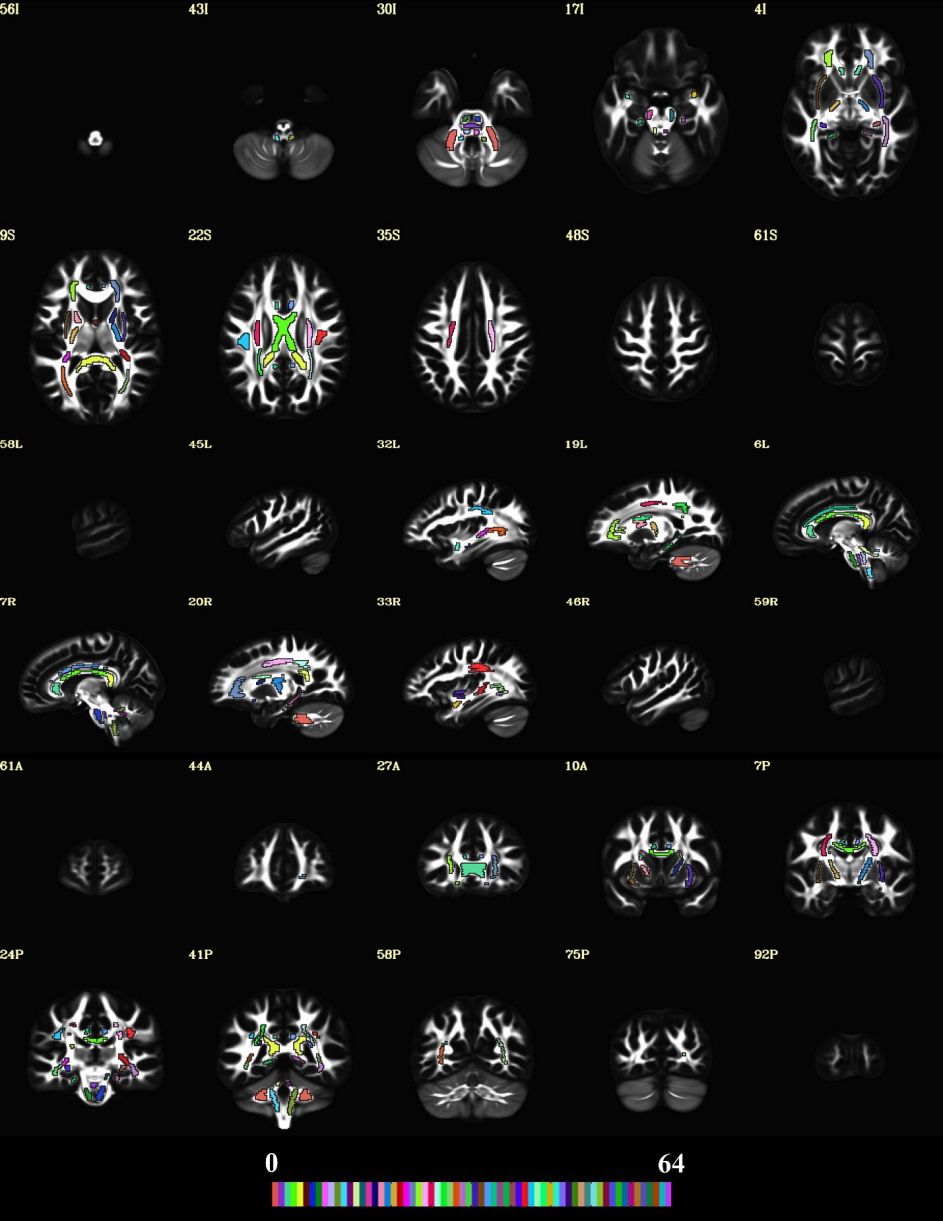
The regions of interest (ROIs) used in the current study. A 5 × 2 montage was created in all orthogonal planes to show the spatial extent of the ROIs overlaid on the average connectome DT FA template. The 48 ROIs are shown using a colormap with a range of 64 colors using AFNI. The slice labels with the correct direction are provided on the top-left of each image in the montage. The “A”, “P”, “I”, “S”, “L” and “R”, labels represent the anterior, posterior, inferior, superior, left and right directions, respectively.

**Figure B.**
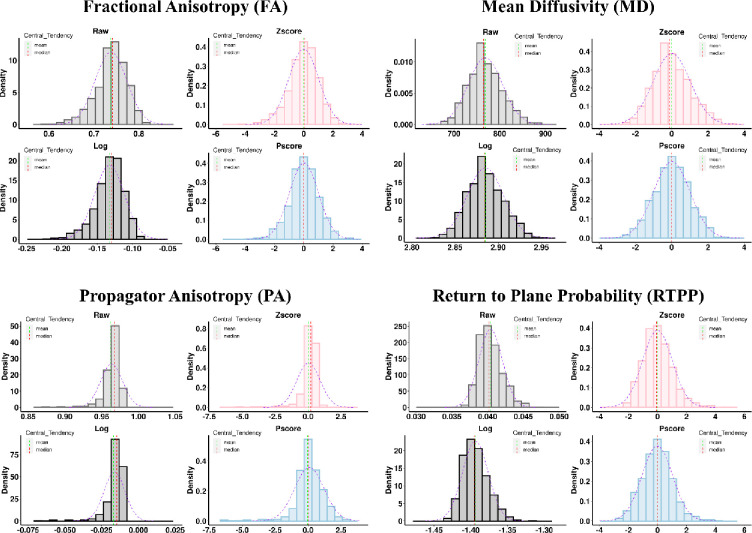
Comparing distributions across different normalization methods for two DT and two MAP metrics from a representative ROI: Body of the Corpus Callosum. The DT metrics are shown in the top row: FA (left) and MD (right); and the MAP metrics are on the bottom row: PA (left) and RTPP (right). For each metric, four distributions are shown. The ‘Raw’ (light gray) distribution panel represents average metric values. The ‘Log’ (dark gray), ‘Zscore’ (light red) and ‘Pscore’ (light blue) distribution panels represent the logarithmic, standardized Z values and the proposed Pscores, respectively. FA (top left) and PA (bottom left) are negatively skewed; however, PA is more heavy-tailed and shows greater skewness. The green and red dashed lines represent the mean and median of the distributions. For both FA and PA, the ‘mean’ underestimates the most common values and appears before the median and the mode of the distribution for ‘Raw’, ‘Log’ and ‘Zscore’ panels. However, the ‘Pscore’ panel shows that all three central tendencies coincide nicely and attain a closer fit to the normal density curve (dashed purple). On the other hand, MD (top right) and RTPP (bottom right) are positively skewed and the ‘mean’ overestimates the most common values for ‘Raw’, ‘Log’ and ‘Zscore’ panels, but the ‘Pscore’ panel shows the consistent alignment of the mean, mode and median and a closer fit to the normal density curve.

**Figure C.**
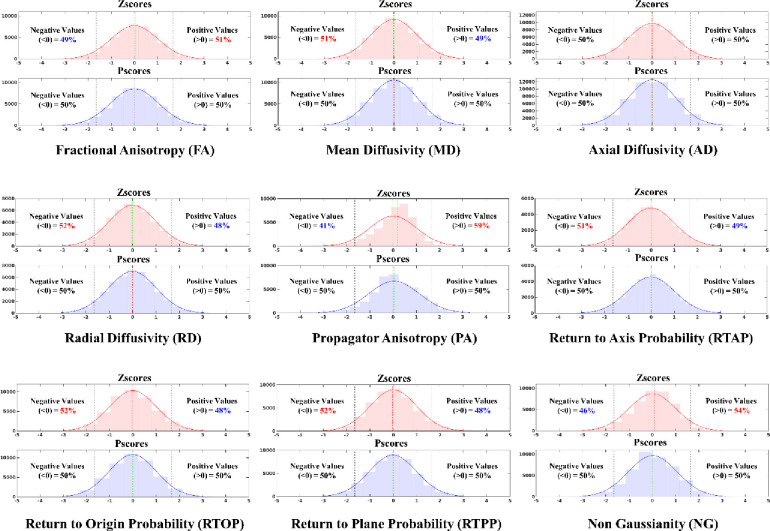
Comparing distributions of Zscores against Pscores across all WM ROIs per dMRI metric. For each dMRI metric, a pair of distributions are shown – one with Zscore (top, light red) histograms and one for Pscore (bottom, light blue) histograms. The values in these distributions come from concatenating all the scores across all ROIs into a single vector. It was done to show if there is an overall bias across the entire WM. A normal density curve (solid red and blue) was also fit on top of each distribution, respectively, to assess which normalization method attained a closer fit to a Gaussian distribution. Except for AD, where the Zscore distribution from the overall WM was approximately normal, an imbalance of negative and positive scores was observed on the distributions from all other dMRI metrics. Zscore distributions for FA, PA and NG show an overall negative skew with less negative (< 50%) and more positive (> 50%) values from the zero line (mean), whereas Pscores maintain a balanced number of negative and positive values (50% on both sides). On the contrary, Zscores from MD, RD, RTAP, RTOP and RTPP show an overall positive skew, with more negative (> 50%) and less positive (< 50%) values around the zero line. Pscores, however, consistently maintain an equal distribution of 50% negative and positive values on either side.

**Figure D.**
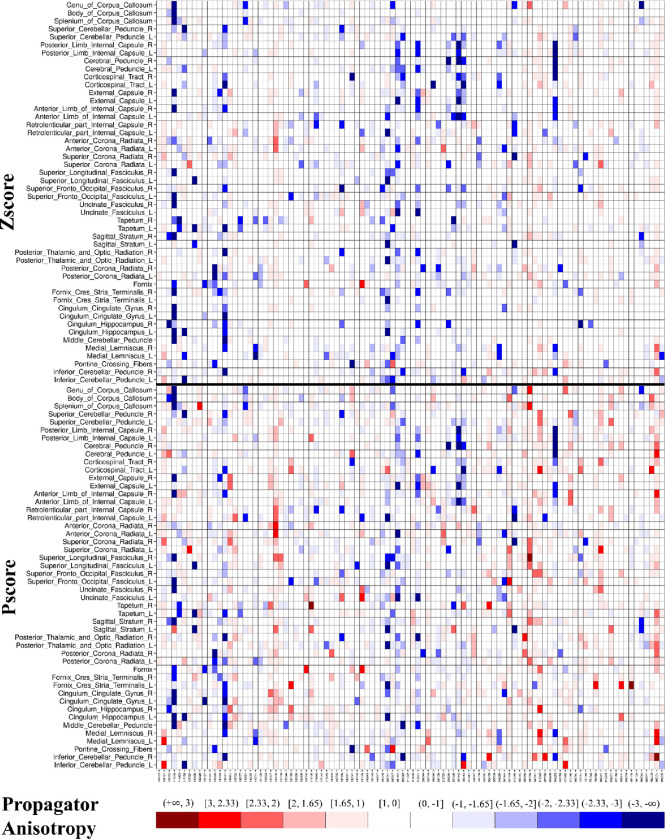
Heatmap comparing Zscores and Pscores from a single iteration of randomly selected sample of 100 participants for the Propagator Anisotropy. The horizontal black line separates the Zscores (top) from the Pscores (bottom). The columns represent individual participants, and the rows represent the regions of interest (ROIs). The ‘R’ and ‘L’ in the ROI names on the y-axis represent the right and left side of the brain, respectively. The x-axis shows the labels of the 100 participants randomly selected from the HCP. The range of Pscores are shown in the colorbar. The parenthesis ‘(‘ in a colorbar tile means that the corresponding shade is exclusive of the value next to it, whereas the bracket ‘]’ means the value is inclusive. For example, the ‘[1.65,1)’ label on top of the lightest shade of red means it represents values in the range ‘1 > Z ≤ 1.65’ and so on for others. Darker shades of blue and red take on extreme negative and positive values, respectively. An individual with a dark blue tile with the range label (−3, −∞) would correspond to Z < −3 from a normal distribution and vice-versa for a dark red shade. The negative extreme value bias in Zscores (top) is quite conspicuous and a balanced positive and negative extreme values in the Pscores (bottom) is evident just from visual inspection.

**Table1.** Assessing Extreme Value Bias in Zscores Compared to Pscores

Metric	Method	N_>95_	N_<5_	N_Total_	P_>95_(%)	P_<5_(%)

	Zscore	1873	2255	46080	4.1	4.9
**FA**						
	Pscore	2304	2304	46080	5.0	5.0
	Zscore	2383	2020	46080	5.2	4.4
**MD**						
	Pscore	2304	2303	46080	5.0	5.0
	Zscore	2117	2183	46080	4.6	4.7
**AD**						
	Pscore	2305	2304	46080	5.0	5.0
	Zscore	2423	1907	46080	5.3	4.1
**RD**						
	Pscore	2304	2304	46080	5.0	5.0
	Zscore	450	2527	43776	1.0	5.8
**PA**						
	Pscore	2208	2208	43776	5.0	5.0
	Zscore	2255	2030	43776	5.2	4.6
**RTAP**						
	Pscore	2208	2208	43776	5.0	5.0
	Zscore	2348	1831	43776	5.4	4.2
**RTOP**						
	Pscore	2208	2208	43776	5.0	5.0
	Zscore	2419	1753	43776	5.5	4.0
**RTPP**						
	Pscore	2208	2208	43776	5.0	5.0
	Zscore	1244	2364	43776	2.8	5.4
**NG**						
	Pscore	2208	2208	43776	5.0	5.0

The **N_>95_** and **P_>95_(%)** show the number and percentage of extreme values, respectively, above the 95^th^ percentile edge value of Z = 1.645 from a normal distribution. On the other hand, the **N_<5_** and **P_<5_(%)** show the number and percentage of extreme values below the 5^th^ percentile edge Z = −1.645. **N_Total_** is the total number of values across all ROIs in the entire sample. Abbreviations: FA = Fractional Anisotropy, MD = Mean Diffusivity, AD = Axial Diffusivity, RD = Radial Diffusivity, PA = Propagator Anisotropy, RTAP = Return to Axis Probability, RTOP = Return of Origin Probability, RTPP = Return to Plane Probability, NG = Non-Gaussianity

**Table2.** Comparing Extreme Value Bias in Zscores vs. Pscores of Propagator Anisotropy for 20 Iterations of Bootstrapping 100 HCP Participants

Metric	Iteration	Method	N_>95_	N_<5_	N_Total_	P_>95_(%)	P_<5_(%)

Propagator Anisotropy	1	Zscore	56	297	4800	1.2	6.2
		Pscore	240	240	4800	5.0	5.0
Propagator Anisotropy	2	Zscore	80	295	4800	1.7	6.1
		Pscore	240	240	4800	5.0	5.0
Propagator Anisotropy	3	Zscore	60	294	4800	1.3	6.1
		Pscore	240	240	4800	5.0	5.0
Propagator Anisotropy	4	Zscore	83	258	4800	1.7	5.4
		Pscore	240	240	4800	5.0	5.0
Propagator Anisotropy	5	Zscore	78	267	4800	1.6	5.6
		Pscore	240	240	4800	5.0	5.0
Propagator Anisotropy	6	Zscore	63	291	4800	1.3	6.1
		Pscore	240	240	4800	5.0	5.0
Propagator Anisotropy	7	Zscore	70	295	4800	1.5	6.1
		Pscore	240	240	4800	5.0	5.0
Propagator Anisotropy	8	Zscore	80	276	4800	1.7	5.8
		Pscore	240	240	4800	5.0	5.0
Propagator Anisotropy	9	Zscore	75	318	4800	1.6	6.6
		Pscore	240	240	4800	5.0	5.0
Propagator Anisotropy	10	Zscore	55	320	4800	1.1	6.7
		Pscore	240	240	4800	5.0	5.0
Propagator Anisotropy	11	Zscore	57	281	4800	1.2	5.9
		Pscore	240	240	4800	5.0	5.0
Propagator Anisotropy	12	Zscore	60	285	4800	1.3	5.9
		Pscore	240	240	4800	5.0	5.0
Propagator Anisotropy	13	Zscore	49	303	4800	1.0	6.3
		Pscore	240	240	4800	5.0	5.0
Propagator Anisotropy	14	Zscore	80	279	4800	1.7	5.8
		Pscore	240	240	4800	5.0	5.0
Propagator Anisotropy	15	Zscore	74	305	4800	1.5	6.4
		Pscore	240	240	4800	5.0	5.0
Propagator Anisotropy	16	Zscore	70	293	4800	1.5	6.1
		Pscore	240	240	4800	5.0	5.0
Propagator Anisotropy	17	Zscore	81	290	4800	1.7	6.0
		Pscore	240	240	4800	5.0	5.0
Propagator Anisotropy	18	Zscore	69	282	4800	1.4	5.9
		Pscore	240	240	4800	5.0	5.0
Propagator Anisotropy	19	Zscore	72	294	4800	1.5	6.1
		Pscore	240	240	4800	5.0	5.0
Propagator Anisotropy	20	Zscore	75	303	4800	1.6	6.3
		Pscore	240	240	4800	5.0	5.0

Same descriptions as Table1 apply. For Zscores, the mean **P_<5_(%)** = 6.1±0.3% and that of **P_>95_(%)** = 1.4±0.2%. For Pscores, the **P_<5_(%)** = **P_>95_(%)** = 5.0±0%.

## References

[1] Fraza CJ, Dinga R, Beckmann CF, Marquand AF. Warped Bayesian linear regression for normative modelling of big data. NeuroImage 2021;245:118715. 34798518 10.1101/2021.04.05.438429 PMC7613680

[2] Kjelkenes R, Wolfers T, Alnæs D, Deviations from normative brain white and gray matter structure are associated with psychopathology in youth. Dev Cogn Neurosci 2022;58:101173. 36332329 10.1016/j.dcn.2022.101173 PMC9637865

[3] Lv J, Di Biase M, Cash RFH, Individual deviations from normative models of brain structure in a large cross-sectional schizophrenia cohort. Molecular Psychiatry 2021;26(7):3512–3523. 32963336 10.1038/s41380-020-00882-5 PMC8329928

[4] Marquand AF, Rezek I, Buitelaar J, Beckmann CF. Understanding Heterogeneity in Clinical Cohorts Using Normative Models: Beyond Case-Control Studies. Biol Psychiatry 2016;80(7):552–561. 26927419 10.1016/j.biopsych.2015.12.023 PMC5023321

[5] Shirk SD, Mitchell MB, Shaughnessy LW, A web-based normative calculator for the uniform data set (UDS) neuropsychological test battery. Alzheimers Res Ther 2011;3(6):32. 22078663 10.1186/alzrt94 PMC3308021

[6] Sherwood B, Zhou AX, Weintraub S, Wang L. Using quantile regression to create baseline norms for neuropsychological tests. Alzheimers Dement (Amst) 2016;2:12–18. 27239531 10.1016/j.dadm.2015.11.005 PMC4879644

[7] Koenker R, Bassett G. Regression Quantiles. Econometrica 1978;46(1):33–50.

[8] Basser PJ, Mattiello J, LeBihan D. MR diffusion tensor spectroscopy and imaging. Biophys J 1994;66(1):259–267. 8130344 10.1016/S0006-3495(94)80775-1 PMC1275686

[9] Pierpaoli C, Jezzard P, Basser PJ, Barnett A, Di Chiro G. Diffusion tensor MR imaging of the human brain. Radiology 1996;201(3):637–648. 8939209 10.1148/radiology.201.3.8939209

[10] Özarslan E, Koay CG, Shepherd TM, Mean apparent propagator (MAP) MRI: a novel diffusion imaging method for mapping tissue microstructure. NeuroImage 2013;78:16–32. 23587694 10.1016/j.neuroimage.2013.04.016 PMC4059870

[11] Hafiz R, Nayak A, Irfanoglu MO, Chan L, Pierpaoli C. Using ‘P-scores’: a novel percentile-based normalization method to accurately assess individual deviation in heavily skewed neuroimaging data. 2023 ISMRM & ISMRT Annual Meeting & Exhibition, Toronto, Canada, Program Abstract Number #3781, ISSN# 1545–4428 | Published date: 19 May, 2023.

[12] Sudlow C, Gallacher J, Allen N, UK biobank: an open access resource for identifying the causes of a wide range of complex diseases of middle and old age. PLoS Med 2015;12(3):e1001779. 25826379 10.1371/journal.pmed.1001779 PMC4380465

[13] Feng C, Wang H, Lu N, Log-transformation and its implications for data analysis. Shanghai Arch Psychiatry 2014;26(2):105–109. 25092958 10.3969/j.issn.1002-0829.2014.02.009 PMC4120293

[14] Robert CP, Casella G, Casella G. Monte Carlo statistical methods: Springer: 1999.

[15] Van Essen DC, Ugurbil K, Auerbach E, The Human Connectome Project: a data acquisition perspective. NeuroImage 2012;62(4):2222–2231. 22366334 10.1016/j.neuroimage.2012.02.018 PMC3606888

[16] Irfanoglu MO, Nayak A, Taylor P, Pierpaoli C. TORTOISE V4: ReImagining the NIH Diffusion MRI Processing Pipeline. 2023 ISMRM & ISMRT Annual Meeting & Exhibition, Toronto, Canada, Program Abstract Number #0080.

[17] WU-Minn H. 1200 subjects data release reference manual. URL https://www.humanconnectome.org. 2017;565.

[18] Irfanoglu MO, Nayak A, Jenkins J, Pierpaoli C. TORTOISE v3: Improvements and New Features of the NIH Diffusion MRI Processing Pipeline. ISMRM, 2018. 2018.

[19] Veraart J, Fieremans E, Novikov DS. Diffusion MRI noise mapping using random matrix theory. Magnetic resonance in medicine 2016;76(5):1582–1593. 26599599 10.1002/mrm.26059 PMC4879661

[20] Kellner E, Dhital B, Kiselev VG, Reisert M. Gibbs-ringing artifact removal based on local subvoxel-shifts. Magnetic resonance in medicine 2016;76(5):1574–1581. 26745823 10.1002/mrm.26054

[21] Irfanoglu MO, Modi P, Nayak A, Hutchinson EB, Sarlls J, Pierpaoli C. DR-BUDDI (Diffeomorphic Registration for Blip-Up blip-Down Diffusion Imaging) method for correcting echo planar imaging distortions. NeuroImage 2015;106:284–299. 25433212 10.1016/j.neuroimage.2014.11.042 PMC4286283

[22] Gu X, Eklund A. Evaluation of Six Phase Encoding Based Susceptibility Distortion Correction Methods for Diffusion MRI. Front Neuroinform 2019;13:76. 31866847 10.3389/fninf.2019.00076 PMC6906182

[23] Schilling KG, Blaber J, Huo Y, Synthesized b0 for diffusion distortion correction (Synb0-DisCo). Magnetic resonance imaging 2019;64:62–70. 31075422 10.1016/j.mri.2019.05.008 PMC6834894

[24] Vos SB, Tax CM, Luijten PR, Ourselin S, Leemans A, Froeling M. The importance of correcting for signal drift in diffusion MRI. Magnetic resonance in medicine 2017;77(1):285–299. 26822700 10.1002/mrm.26124

[25] Rudrapatna U, Parker GD, Roberts J, Jones DK. A comparative study of gradient nonlinearity correction strategies for processing diffusion data obtained with ultra-strong gradient MRI scanners. Magnetic resonance in medicine 2021;85(2):1104–1113. 33009875 10.1002/mrm.28464 PMC8103165

[26] Irfanoglu MO, Nayak A, Jenkins J, DR-TAMAS: Diffeomorphic Registration for Tensor Accurate Alignment of Anatomical Structures. NeuroImage 2016;132:439–454. 26931817 10.1016/j.neuroimage.2016.02.066 PMC4851878

[27] Nayak A, Irfanoglu MO, Pierpaoli C. Diffusion MRI Atlases from the Human Connectome Project Data. In Proceedings of International Society of Magnetic Resonance in Medicine; 2020, p.3751.

[28] Basser PJ, Pierpaoli C. Microstructural and physiological features of tissues elucidated by quantitative-diffusion-tensor MRI. J Magn Reson B 1996;111(3):209–219. 8661285 10.1006/jmrb.1996.0086

[29] Oishi K, Zilles K, Amunts K, Human brain white matter atlas: identification and assignment of common anatomical structures in superficial white matter. NeuroImage 2008;43(3):447–457. 18692144 10.1016/j.neuroimage.2008.07.009 PMC2586008

[30] Yushkevich PA, Piven J, Hazlett HC, User-guided 3D active contour segmentation of anatomical structures: significantly improved efficiency and reliability. NeuroImage 2006;31(3):1116–1128. 16545965 10.1016/j.neuroimage.2006.01.015

[31] Irfanoglu MO, Beyh A, Catani M, Dell’Acqua F, Pierpaoli C. ReImagining the Young Adult Human Connectome Project (HCP) Diffusion MRI Dataset. Proceedings of the International Society for Magnetic Resonance in Medicine 30; 2022.

[32] Nayak A, Walker L, Pierpaoli C, and The Brain Development Cooperative Group. Evaluation of pre-defined atlas based ROIs for the analysis of DTI data in Normal Brain Development. Proc. Intl. Soc. Mag. Reson. Med. 20. 2012.

[33] Cox SR, Ritchie SJ, Tucker-Drob EM, Ageing and brain white matter structure in 3,513 UK Biobank participants. Nat Commun 2016;7:13629. 27976682 10.1038/ncomms13629 PMC5172385

[34] Pierpaoli C, Jones DK. Removing CSF Contamination in Brain DT-MRIs by Using a Two-Compartment Tensor Model. Proceedings of the International Society for Magnetic Resonance in Medicine 11; 2004.

[35] Pierpaoli C, Jezzard P, Basser PJ, Barnett A, Chiro GD. Diffusion tensor MR imaging of the human brain. Radiology 1996;201(3):637–648. 8939209 10.1148/radiology.201.3.8939209

[36] Filippone M, Engler R. Enabling scalable stochastic gradient-based inference for Gaussian processes by employing the Unbiased LInear System SolvEr (ULISSE). International Conference on Machine Learning: PMLR; 2015. p. 1015–1024.

[37] Saatçi Y. Scalable inference for structured Gaussian process models. Citeseer; 2012.

[38] Snelson E, Ghahramani Z. Sparse Gaussian Processes using Pseudo-inputs. Neural Information Processing Systems; 2005.

[39] Marek S, Tervo-Clemmens B, Calabro FJ, Reproducible brain-wide association studies require thousands of individuals. Nature 2022;603(7902):654–660. 35296861 10.1038/s41586-022-04492-9 PMC8991999

[40] Bishop CM. Pattern Recognition and Machine Learning: All “just the Facts 101” Material: Springer (India) Private Limited: 2013.

[41] Coles S, Bawa J, Trenner L, Dorazio P. An introduction to statistical modeling of extreme values: Springer: 2001.

[42] Davison AC. Extreme values. Encyclopedia of Biostatistics 2005;4.

[43] Ziegler G, Ridgway GR, Dahnke R, Gaser C. Individualized Gaussian process-based prediction and detection of local and global gray matter abnormalities in elderly subjects. NeuroImage 2014;97:333–348. 24742919 10.1016/j.neuroimage.2014.04.018 PMC4077633

