# Supplementary material for: ‘Pscore’ - A Novel Percentile-Based Metric to Accurately Assess Individual Deviations in Non-Gaussian Distributions of Quantitative MRI Metrics
Source: bioRxiv. 2023 Dec 10:2023.12.10.571016. Preprint. [Version 1] doi: 10.1101/2023.12.10.571016 (PMC10723480; doi:10.1101/2023.12.10.571016)
Supplement: Supplementary file 1 [file media-1.pdf]

SUPPLEMENTARY APPENDIX

‘Pscore’ - A Novel Percentile-Based Metric to Accurately Assess Individual Deviations in Non-Gaussian Distributions of Quantitative MRI Metrics.

Table S1. List of Regions of Interest (ROIs) Used in the Current Study

ROI Numbers	ROI Names	Number of Voxels	Volume of ROI /mm^3
1	Middle Cerebellar Peduncle	2320	2320
2	Pontine Crossing Fibers	480	480
3	Genu of Corpus Callosum	1285	1285
4	Body of Corpus Callosum	3283	3283
5	Splenium of Corpus Callosum	3432	3432
6	Fornix	87	87
7	Right Corticospinal Tract	344	344
8	Left Corticospinal Tract	253	253
9	Right Medial Lemniscus	188	188
10	Left Medial Lemniscus	163	163
11	Right Inferior Cerebellar Peduncle	428	428
12	Left Inferior Cerebellar Peduncle	372	372
13	Right Superior Cerebellar Peduncle	179	179
14	Left Superior Cerebellar Peduncle	166	166
15	Right Cerebral Peduncle	333	333
16	Left Cerebral Peduncle	317	317
17	Right Anterior Limb of Internal Capsule	490	490
18	Left Anterior Limb of Internal Capsule	572	572
19	Right Posterior Limb of Internal Capsule	832	832
20	Left Posterior Limb of Internal Capsule	624	624
21	Right Retrolenticular part of Internal Capsule	614	614
22	Left Retrolenticular part of Internal Capsule	466	466
23	Right Anterior Corona Radiata	1529	1529
24	Left Anterior Corona Radiata	1319	1319
25	Right Superior Corona Radiata	1934	1934
26	Left Superior Corona Radiata	1343	1343
27	Right Posterior Corona Radiata	586	586
28	Left Posterior Corona Radiata	630	630
29	Right Posterior Thalamic and Optic Radiation	660	660
30	Left Posterior Thalamic and Optic Radiation	806	806
31	Right Sagittal Stratum	938	938
32	Left Sagittal Stratum	602	602
33	Right External Capsule	1461	1461
34	Left External Capsule	921	921
35	Right Cingulum Cingulate Gyrus	758	758
36	Left Cingulum Cingulate Gyrus	649	649
37	Right Cingulum Hippocampus	324	324
38	Left Cingulum Hippocampus	261	261
39	Right Fornix Cres Stria Terminalis	184	184
40	Left Fornix Cres Stria Terminalis	192	192

ROI Numbers	ROI Names	Number of Voxels	Volume of ROI /mm^3
41	Right Superior Longitudinal Fasciculus	1047	1047
42	Left Superior Longitudinal Fasciculus	886	886
43	Right Superior Fronto Occipital Fasciculus	100	100
44	Left Superior Fronto Occipital Fasciculus	80	80
45	Right Uncinate Fasciculus	123	123
46	Left Uncinate Fasciculus	83	83
47	Right Tapetum	74	74
48	Left Tapetum	86	86

Footnote: The **'Number of Voxels'** represents the total sum of the number of voxels within an ROI. The **'Volume of ROI'** represents the total volume in mm^3 of each ROI.

Table S2. Assessing Extreme Value Bias in Zscores vs Pscores of all Diffusion MRI Metrics from Bootstrapping Across 100 Iterations.

Metric	Method	$\overline{\mathbf{N}_{>95}}$	$\overline{\mathbf{N}_{<5}}$	$\overline{\mathbf{N}_{Total}}$	$\overline{P_{>95}}$(%) Mean ± SD	$\overline{P_{<5}}$(%) Mean ± SD
Fractional Anisotropy (FA)	Zscore	206	246	4800	4.3 ± 0.4	5.1 ± 0.3
	Pscore	240	240	4800	5.0 ± 0.0	5.0 ± 0.0
Mean Diffusivity (MD)	Zscore	254	219	4800	5.3 ± 0.3	4.6 ± 0.3
	Pscore	240	240	4800	5.0 ± 0.0	5.0 ± 0.0
Axial Diffusivity (AD)	Zscore	228	238	4800	4.8 ± 0.3	5.0 ± 0.3
	Pscore	240	240	4800	5.0 ± 0.0	5.0 ± 0.0
Radial Diffusivity (RD)	Zscore	256	206	4800	5.3 ± 0.3	4.3 ± 0.3
	Pscore	240	240	4800	5.0 ± 0.0	5.0 ± 0.0
Propagator Anisotropy (PA)	Zscore	65	289	4800	1.4 ± 0.2	6.0 ± 0.3
	Pscore	240	240	4800	5.0 ± 0.0	5.0 ± 0.0
Return to Axis Probability (RTAP)	Zscore	252	226	4800	5.3 ± 0.3	4.7 ± 0.3
	Pscore	240	240	4800	5.0 ± 0.0	5.0 ± 0.0
Return to Origin Probability (RTOP)	Zscore	266	211	4800	5.5 ± 0.3	4.4 ± 0.3
	Pscore	240	240	4800	5.0 ± 0.0	5.0 ± 0.0
Return to Plane Probability (RTPP)	Zscore	268	198	4800	5.6 ± 0.2	4.1 ± 0.3
	Pscore	240	240	4800	5.0 ± 0.0	5.0 ± 0.0
Non Gaussianity (NG)	Zscore	151	271	4800	3.1 ± 0.3	5.6 ± 0.2
	Pscore	240	240	4800	5.0 ± 0.0	5.0 ± 0.0

Footnote: The $\overline{\mathbf{N}_{>95}}$ and $\overline{P_{>95}}$ show the mean number and percentage of extreme values, respectively, above the 95th percentile edge value of Z = 1.645 from a normal distribution. The 'accent' bar on top represents the 'mean' across 100 iterations. The $\overline{\mathbf{N}_{<5}}$ and $\overline{P_{<5}}$ show the mean number and percentage of extreme values below the 5th percentile edge value of Z = -1.645. $\overline{\mathbf{N}_{Total}}$ is the mean across 100 iterations for the total number of values across all ROIs in the randomly selected sample of 100 participants. For the $\overline{P_{>95}}$ and $\overline{P_{<5}}$ values**,** both the mean and standard deviation (SD) across 100 iterations, are shown for each method.

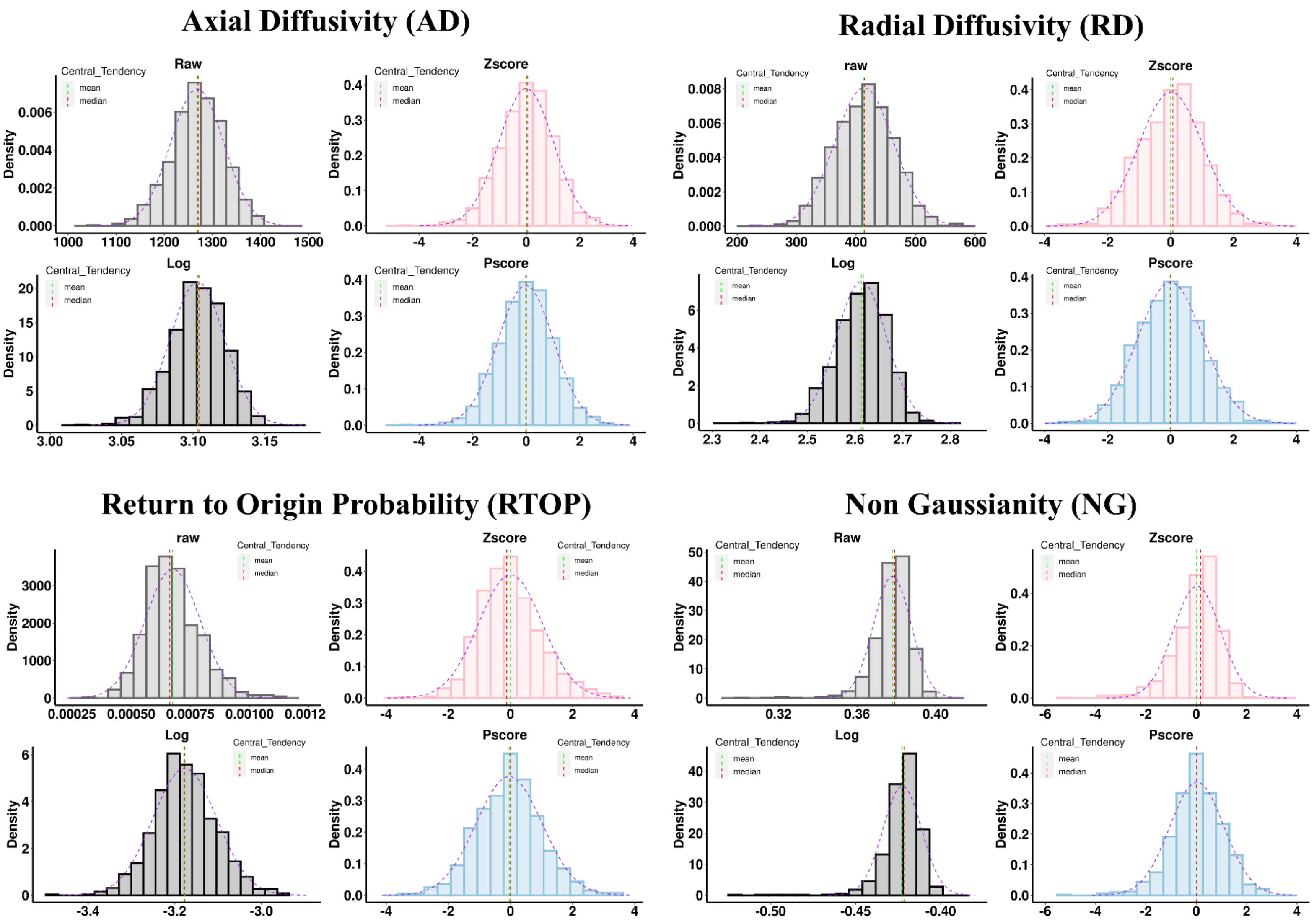

Figure SA. Comparing distributions across different normalization methods for two DT and two MAP metrics from a representative ROI: Right Anterior Limb of the Internal Capsule. The DT metrics are shown in the top row: AD (left) and RD

(right); and the MAP metrics are on the bottom row: RTOP (left) and NG (right). For each metric, four distributions are shown. The ‘Raw’ (light gray) distribution panel represents average metric values. The ‘Log’ (dark gray), ‘Zscore’ (light red) and ‘Pscore’ (light blue) distribution panels represent the logarithmic, standardized Z values and the proposed Pscores, respectively. AD (top left), RD (top right) and NG (bottom right) are negatively skewed, with NG showing the strongest skew. The green and red dashed lines represent the mean and median of the distributions. For FA, RD and NG, the ‘mean’ underestimates the most common values and appears before the median and the mode of the distribution for ‘Raw’, ‘Log’ and ‘Zscore’ panels. However, the ‘Pscore’ panel shows that all three moments align nicely and attain a closer fit to the normal density curve (dashed purple). On the other hand, RTOP (bottom left) is positively skewed and the ‘mean’ overestimates the most common values for ‘Raw’, ‘Log’ and ‘Zscore’ panels, but the ‘Pscore’ panel shows a closer alignment of mean, mode and median and a better fit to the normal density curve.

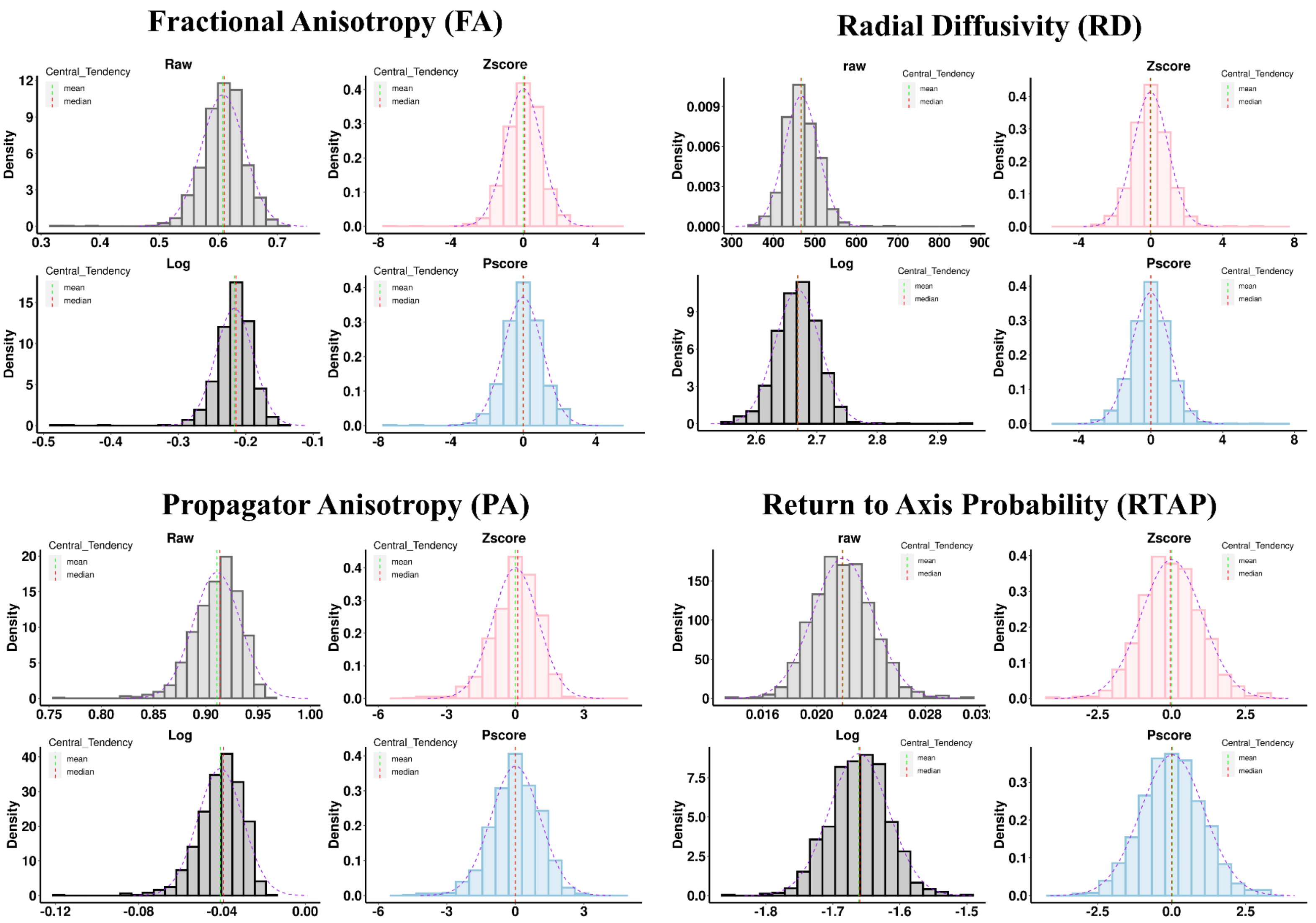

Figure SB. Comparing distributions across different normalization methods for two DT and two MAP metrics from a representative ROI: Left Medial Lemniscus. Similar descriptions as Figure SA applies except for DT metrics – FA (top left) and RD

(top right); and MAP metrics – PA (bottom left) and RTAP (bottom right). FA, PA and RTAP are negatively skewed. For these metrics, the 'mean' underestimates the most common values and appears before the median and the mode of the distribution for 'Raw', 'Log' and 'Zscore' panels. However, the 'Pscore' panel shows that all three moments align nicely and attain a closer fit to the normal density curve (dashed purple). On the other hand, RD is positively skewed and the 'mean' overestimates the most common values for 'Raw', 'Log' and 'Zscore' panels. However, the 'Pscore' panel shows the consistent alignment of the mean, mode and median and a closer fit to the normal density curve.
